# Titanium dioxide nanoparticles induce the expression of early and late receptors for adhesion molecules on monocytes

**DOI:** 10.1186/s12989-016-0147-3

**Published:** 2016-06-23

**Authors:** Cristhiam Rueda-Romero, Guillermina Hernández-Pérez, Pilar Ramos-Godínez, Inés Vázquez-López, Raúl Omar Quintana-Belmares, Elizabeth Huerta-García, Ewa Stepien, Rebeca López-Marure, Angélica Montiel-Dávalos, Ernesto Alfaro-Moreno

**Affiliations:** 1 Environmental Toxicology Laboratory, Subdirección de Investigación Básica, Instituto Nacional de Cancerología, Ciudad de México, México; 2 Universidad Interserrana del Estado de Puebla, Ahuacatlán, Puebla México; 3 Electron Microscopy Laboratory, Subdirección de Patología, Instituto Nacional de Cancerología, Ciudad de México, México; 4 Cell Biology Laboratory, Physiology Department, Instituto Nacional de Cardiología, Ciudad de México, México; 5 M. Smoluchowski Institute of Physics, Jagiellonian University, Krakow, Poland; 6 Swedish Toxicology Sciences Research Center (Swetox), Forskargatan 20, 151 36 Södertälje, Sweden

**Keywords:** Titanium dioxide nanoparticles, Monocyte, Endothelial cell, Adhesion molecules, sLe^x^, PSGL-1, LFA-1, VLA-4, αVβ3

## Abstract

**Background:**

There is growing evidence that exposure to titanium dioxide nanoparticles (TiO_2_ NPs) could be harmful. Previously, we have shown that TiO_2_ NPs induces endothelial cell dysfunction and damage in glial cells. Considering that inhaled particles can induce systemic effects and the evidence that nanoparticles may translocate out of the lungs, we evaluated whether different types of TiO_2_ NPs can induce the expression of receptors for adhesion molecules on monocytes (U937 cell line). We evaluated the role of reactive oxygen spices (ROS) on these effects.

**Methods:**

The expression of receptors for early (sLe^x^ and PSGL-1) and late (LFA-1, VLA-4 and αVβ3) adhesion molecules was evaluated in U937 cells on a time course (3–24 h) using a wide range of concentrations (0.001-100 μg/mL) of three types of TiO_2_ NPs (<25 nm anatase, 50 nm anatase-rutile or < 100 nm anatase). Cells exposed to TNFα were considered positive controls, and unexposed cells, negative controls. In some experiments we added 10 μmolar of N-acetylcysteine (NAC) to evaluate the role of ROS.

**Results:**

All tested particles, starting at a concentration of 0.03 μg/mL, induced the expression of receptors for early and late adhesion molecules. The largest increases were induced by the different molecules after 3 h of exposure for sLe^x^ and PSGL-1 (up to 3-fold of the positive controls) and after 18 h of exposure for LFA-1, VLA-4 and αVβ3 (up to 2.5-fold of the positive controls). Oxidative stress was observed as early as 10 min after exposure, but the maximum peak was found after 4 h of exposure. Adhesion of exposed or unexposed monocytes to unexposed or exposed endothelial cells was tested, and we observed that monocytes cells adhere in similar amounts to endothelial cells if one of the two cell types, or both were exposed. When NAC was added, the expression of the receptors was inhibited.

**Conclusions:**

These results show that small concentrations of particles may activate monocytes that attach to endothelial cells. These results suggest that distal effects can be induced by small amounts of particles that may translocate from the lungs. ROS play a central role in the induction of the expression of these receptors.

**Electronic supplementary material:**

The online version of this article (doi:10.1186/s12989-016-0147-3) contains supplementary material, which is available to authorized users.

## Background

Titanium dioxide particles (TiO_2_) have been considered as nontoxic mineral particles and traditionally used in the fields of cosmetics, food, and drugs. They were even used as “dust negative control” in many *in vitro* and *in vivo* toxicological investigations for many years [1, 2]. Titanium dioxide nanoparticles (TiO_2_ NPs) consists of three crystals forms, including anatase, rutile, and brookite [3]. TiO_2_ NPs have been widely used in many products, such as toothpastes, sunscreens, cosmetics, food products, pharmaceuticals, and nanomedical reagents [4]. However, research evidence suggests that TiO_2_ NPs may possess higher toxicity potential than their bulk materials [4–6]. Several investigations found that TiO_2_ NPs can penetrate basic biological structures, which may, in turn, disrupt their normal function [1, 6, 7]. Also recent research evidence shows that TiO_2_ NPs may induce cellular toxicity effects in cardiac tissue [8]. The toxic effects of TiO_2_ NPs were also observed in cells of the circulatory system. Previous studies found that erythrocytes treated with TiO_2_ NPs underwent abnormal sedimentation, hemagglutination, and hemolysis, which were totally different from those treated with fine particles of TiO_2_ [2]. Size, effective cellular dose, biokinetics, physicochemical and surface properties could be responsible for these differences [9].

Exposure to nanoparticles has been linked to local and systemic effects such as lung inflammation, enhanced thrombotic potential and systemic endothelial dysfunction [10]. Increasing amounts of evidence show that TiO_2_ NPs may induce airway irritation, lung inflammation, hepatic and renal effects, proinflammatory effects and systemic microvascular dysfunction [11]. Recently, the International Agency for Research on Cancer (IARC) classified TiO_2_ as a 2B carcinogen [12]. The mechanism by which TiO_2_ NPs induces the above effects is not well understood. Regarding how inhaled nanoparticles or ultrafine particles can induce systemic effects, the hypothesis of particle translocation from the lungs into the bloodstream could explain how an inhaled particle could be linked to a systemic adverse outcome [13–15]. Considering that the alveolar-capillary barrier does not allow large quantities of particles to translocate, it is reasonable to assume that only a tiny fraction of inhaled particles may translocate. Therefore exploring the cellular effects of nanoparticles at very low concentrations is necessary [16, 17].

Several studies have shown *in vivo* and *in vitro* that inhaled particles may induce endothelial activation and dysfunction. *In vitro* evidence indicates that particle concentrations above 1 μg/cm^2^ are needed to induce endothelial dysfunction [18], but it is not clear if these particle concentrations are sufficient for translocation to the endothelium *in vivo*. Few *in vivo* studies showed deleterious effect of TiO_2_ on vascular cells, initiation of endothelial cell dysfunction and damage. Pulmonary exposure (instillation) to high doses of TiO_2_ NPs caused systemic inflammation, dyslipidemia and enhanced atherosclerotic plaque progression in ApoE-knockout mice [19, 20]. However, some contradictory data are also available, showing the modest effect on plaque progression in the same animal model [21]. At cellular level, endothelial dysfunction is associated with local elevation of pro-inflammatory mediators (cytokines, chemoatractants and cell adhesion molecules), which can lead to atherosclerosis, but the role of the local nanoparticle exposure on systemic processes (atherosclerosis) is still poorly understood [8]. Secreted inflammatory factors have been proposed as possible mediators of local and systemic inducers of endothelial dysfunction [22]. Nevertheless, there is no evidence that monocytes may be activated by very small amount of particles. An alternative hypothesis involves the production of circulating microvesicles that may transfer nanoparticles within the bloodstream to reach distant target cells [22].

The monocyte-endothelial cell interaction is mediated by several molecules playing different roles such as capture/tethering, rolling, activation, adhesion, diapedesis, traversing the basal lamina and migration [23]. Previously, we demonstrated that TiO_2_ NPs and urban particles are capable of inducing the expression of E and P selectins and ICAM-1, VCAM-1 and PECAM-1 in endothelial cells exposed to concentrations of 5 μg/cm^2^ [17]. Monocytes express surface molecules that are ligands for adhesion molecules, including sLe^x^ (E-selectin ligand) [24], PSGL-1 (P-selectin ligand) [23], LFA-1 (ICAM-1 ligand) [25], VLA-4 (VCAM-1 ligand) [26] and αVβ3 (PECAM-1 ligand) [27], among others. In the present study, we evaluated whether TiO_2_ NPs activates monocytes and induces the expression of sLe^x^, PSGL-1, LFA-1, VLA-4 and αVβ3, promoting the adhesion of monocytes to endothelial cells, even if these endothelial cells were not challenged with particles.

## Results

All TiO_2_ NPs samples were negative for the presence of endotoxin. The X-RF analysis did not show the presence of any other elements besides oxygen and titanium. The size and ζ-potential for the < 50 nm TiO_2_ NPs have been previously reported [17]. For the < 25 nm TiO_2_ NPs the average size was 19 nm with a ζ-potential of −12 mV. The < 100 nm TiO_2_ NPs average size was 80 nm with a ζ-potential of – 8.9 mV.

The three different sizes of TiO_2_ NPs induced very similar results. To keep the results as simple as possible, we will describe the general results for the < 50 nm anatase-rutile, the effects of which on HUVECs we have previously characterized [17]. The results for < 25 nm anatase and < 100 nm anatase-rutile are presented as Additional file 1.

### Expression of sLe^x^ and PSGL-1

**Fig. 1:**
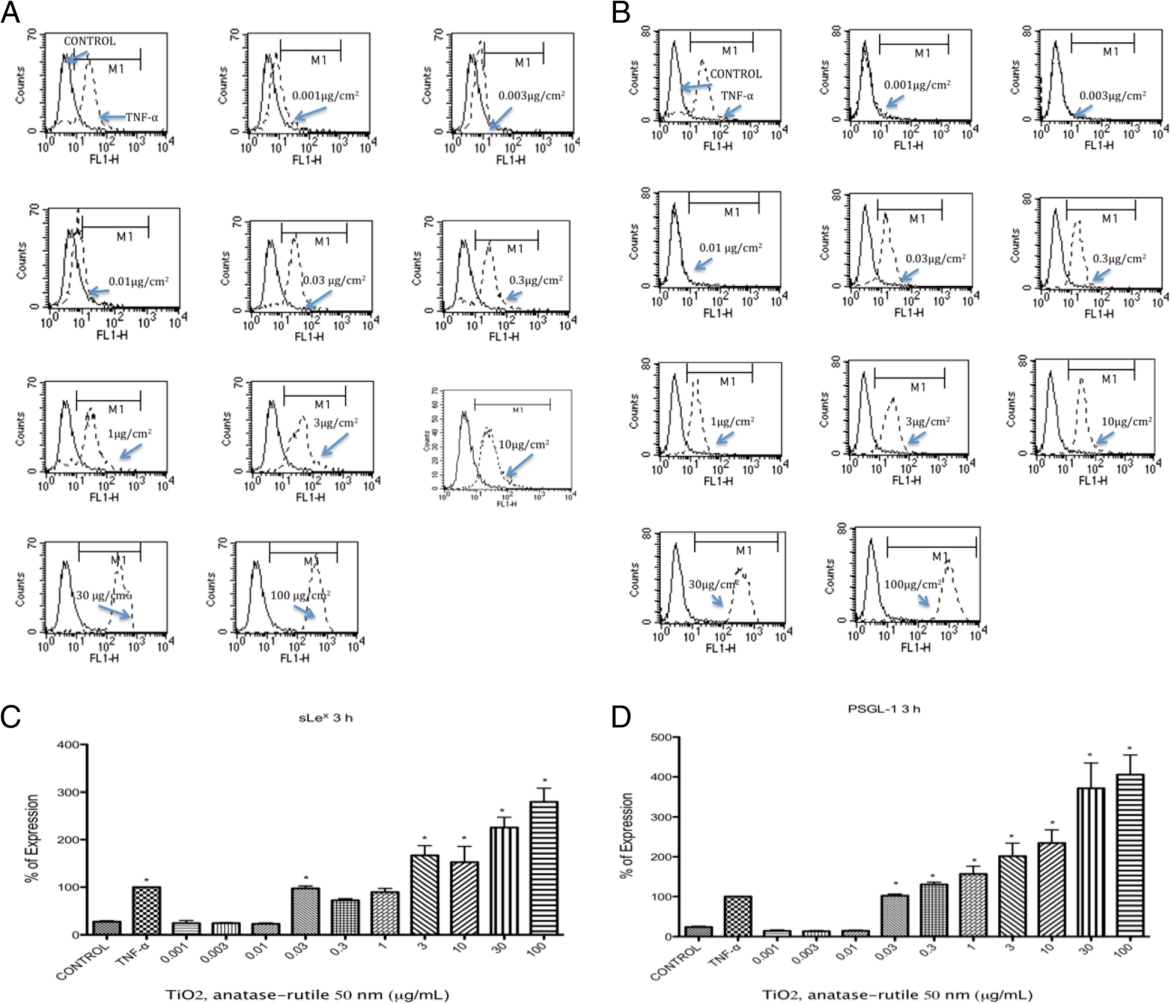
TiO_2_ NPs induced the expression of sLe^x^ and PSGL-1. U937 cells were exposed to TNFα (10 ng/mL) or to concentration of 0.001, 0.003, 0.01, 0.03, 0.3, 1, 3, 10, 30 and 100 μg/mL of TiO_2_ NPs of 50 nm during 3 h. Unexposed cultures were used as negative controls. **a** Histograms of the sLe^x^ expression obtained by flow cytometry. The continuous line represents control unexposed cells and the dotted line corresponds to the different treatments. **b** Histograms of the PSGL-1 expression obtained by flow cytometry. The continuous line represents control unexposed cells and the dotted line corresponds to the different treatments. **c** and **d** Normalization of the results presented as as a percentage of expression (fluorescencence intensity with respect to control) as mean ± SD of three separate experiments. After 3 h of exposure to TiO_2_ NPs an expression of sLe^x^ and PSGL-1 was as strong as the induced by TNFα. *p < 0.05 vs negative control

sLe^x^ and PSGL-1 were induced in U937 cells exposed to TiO_2_ NPs at all times tested (*p* < 0.05 vs negative control). The largest induction was observed after 3 h of exposure (Fig. 1 a-c). Originally, we tested concentrations from 0.3 μg/mL, but due to the absence of negative results with lower concentrations of TiO_2_ NPs, we decided to test even lower concentrations. When concentrations from 0.001 to 0.030 μg/mL were included, we observed negative results at concentrations between 0.001 and 0.010 μg/mL; however, from 0.030 μg/mL, we observed an induction as strong as the induction induced by the positive controls (*p* < 0.05 vs negative control) (Fig. 1 a-d).

### Expression of LFA-1, VLA-4 and αVβ3

**Fig. 2:**
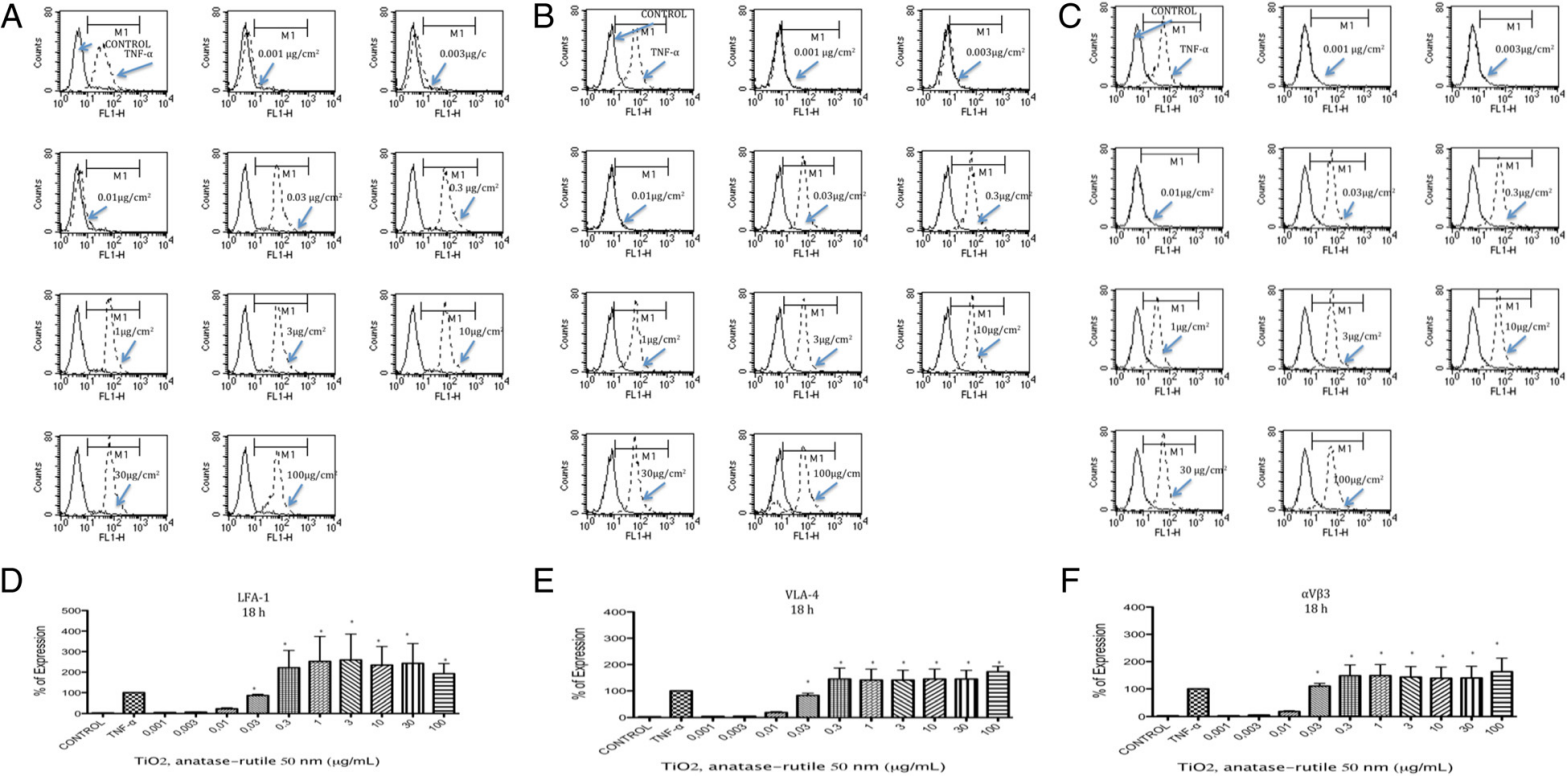
TiO_2_ NPs induced expression of LFA-1, VLA-4 and αVβ3. U937 cells were exposed to TNFα (10 ng/mL) or to concentration of 0.001, 0.003, 0.01, 0.03, 0.3, 1, 3, 10, 30 and 100 μg/mL of TiO_2_ NPs of 50 nm during 18 h. **a** Histograms of the LFA-1 expression obtained by flow cytometry. The continuous line represents control unexposed cells and the dotted line corresponds to the different treatments. **b** Histograms of the VLA-4 expression obtained by flow cytometry. The continuous line represents control unexposed cells and the dotted line corresponds to the different treatments. **c** Histograms of the αVβ3 expression obtained by flow cytometry. The continuous line represents control unexposed cells and the dotted line corresponds to the different treatments. **d**-**f** Normalization of the results presented as as a percentage of expression (fluorescencence intensity with respect to control) as mean ± SD of three separate experiments. After 18 h of exposure to TiO_2_ NPs an expression of sLe^x^ and PSGL-1 was as strong as the induced by TNFα. *p < 0.05 vs negative control

LFA-1, VLA-4 and αVβ3 expression was induced by TiO_2_ NPs at all tested times. The largest amount of expression was observed at 18 h after exposure (Fig. 2 a-f), but levels of expression similar to those of the positive controls were observed as early as 8 h and as late as 24 h after exposure (Additional file 1). Concentrations from 0.001 to 0.01 μg/mL did not induce the expression of LFA-1, VLA-4 or αVβ3, but concentrations at 0.03 μg/mL and above did induce the expression of these molecules (*p* < 0.05 vs negative control) (Fig. 2).

### Adhesion assay

**Fig. 3:**
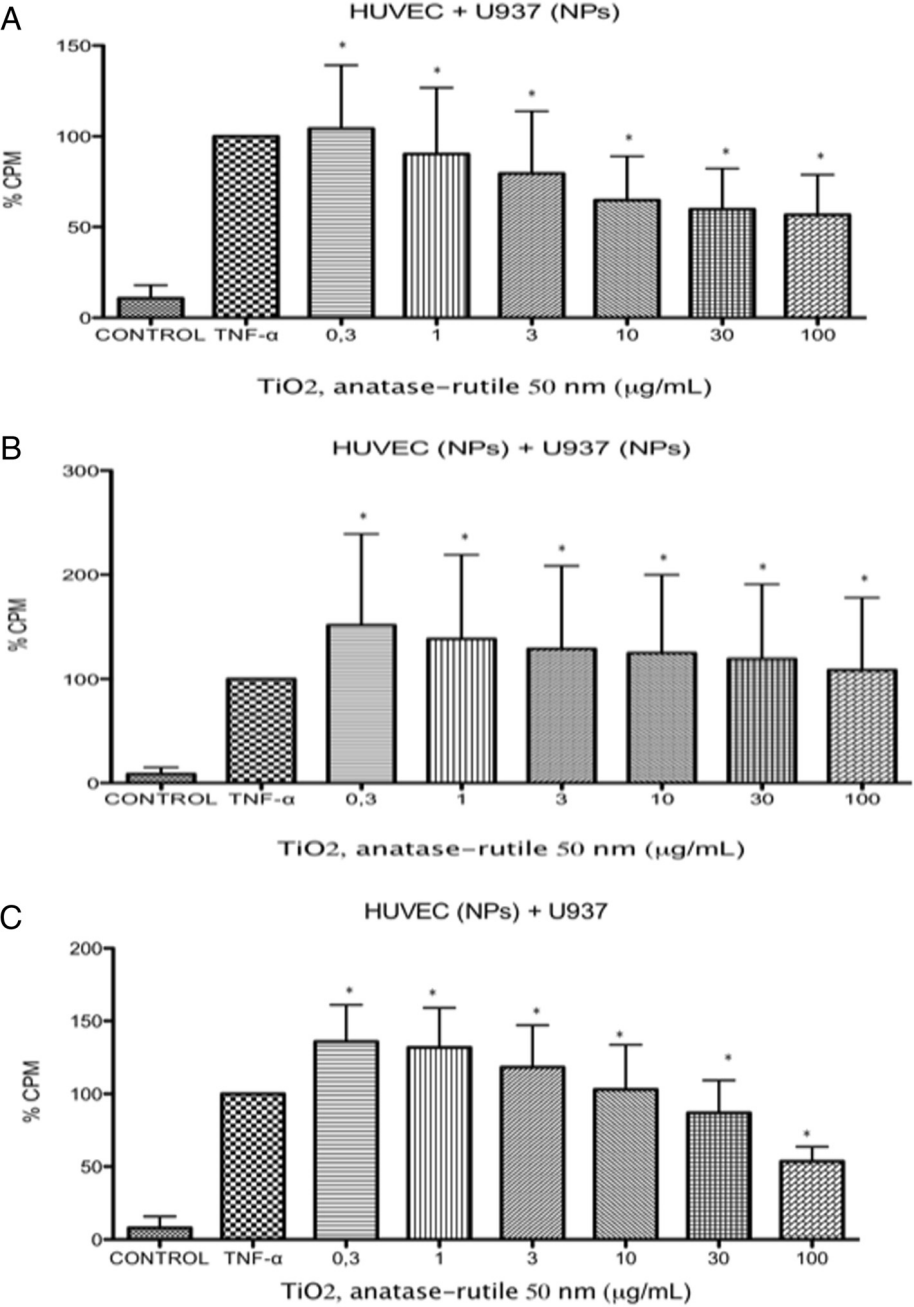
Adhesion of U937 cells to HUVECs. U937 cells were exposed to TNFα (10 ng/mL) or to concentration of 0.3, 1, 3, 10, 30 and 100 μg/mL of TiO_2_ NPs of 50 nm during 3 h. Unexposed cultures were used as negative controls. After 3 h of exposure, the cells were co-cultured with HUVEC that may be naive or exposed to TiO_2_ NPs. **a** U937 cells exposed to TiO_2_ NPs and naive HUVECs; **b** HUVEC and U937 cells exposed to the same concentrations of TiO_2_-NP; **c** HUVEC exposed to TiO_2_-NP and naive U937 cells. The results were similar under all experimental conditions. * *p* < 0.05 vs negative control

When U937 cells were exposed to TiO_2_ NPs, the cells became adherent to naive HUVECs at all evaluated concentrations. The numbers of adherent cells induced were not different than the numbers induced by the positive controls (*p* < 0.05 vs negative control) (Fig. 3). Similar results were observed when the U937 cells and HUVECs were exposed to the same concentrations of TiO_2_ NPs (*p* < 0.05 vs negative control) (Fig. 3). If the HUVECs were exposed and the U937 cells were not exposed, there was no difference in the amount of adhesion induced (*p* < 0.05 vs negative control) (Fig. 3).

### Reactive oxygen species

**Fig. 4:**
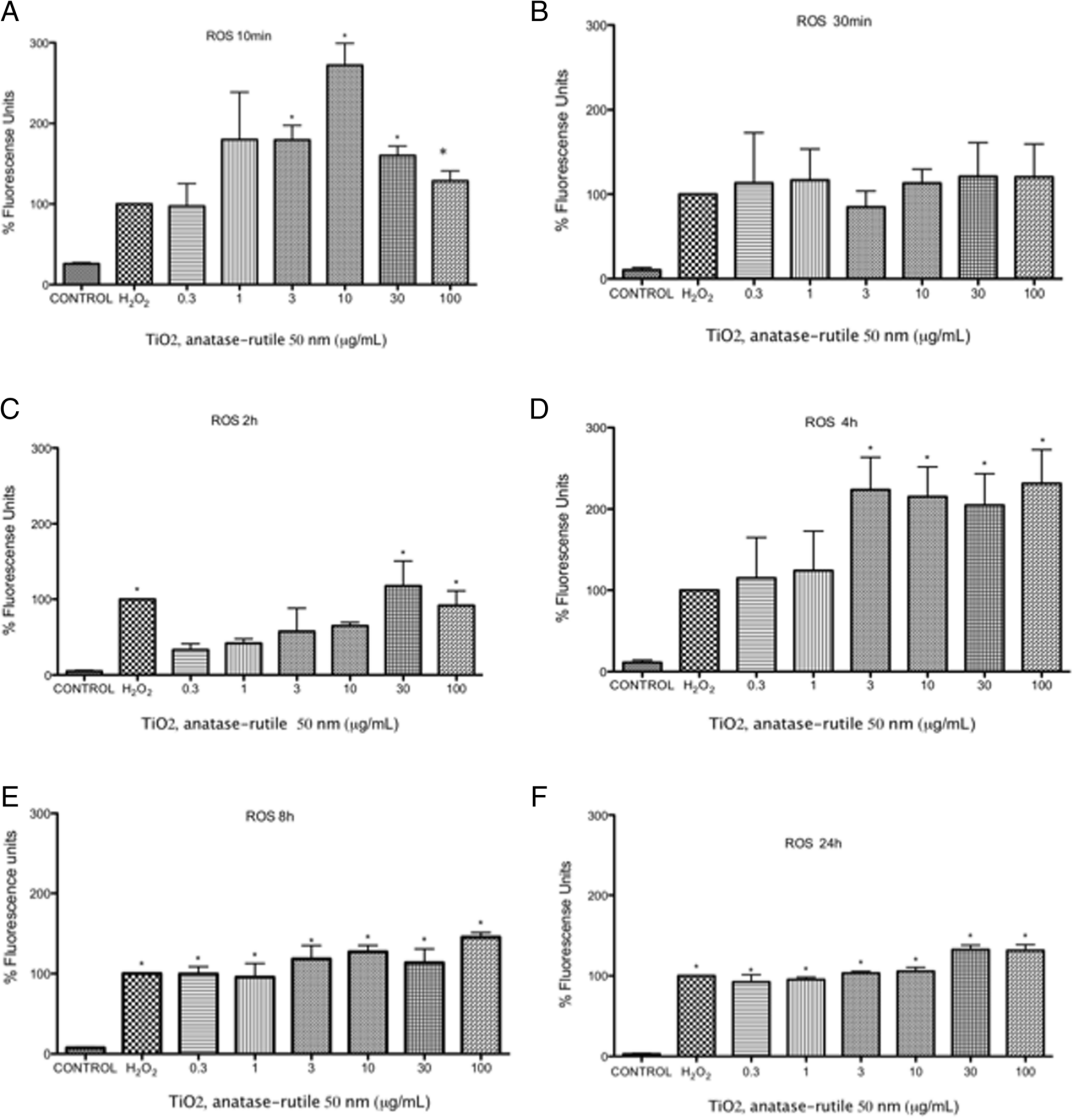
TiO_2_ NPs induced an increase in oxygen reactive species production at different times. **a-f** U937 cells were culture with 0.3, 1, 3, 10, 30 and 100 μg/mL of TiO_2_ NPs of 50 nm for 10, 30 min, 2, 4, 8, 24 h respectively and H_2_0_2_ (300 μM, positive control) or without treatment (negative control). ROS concentration was evaluated using H_2_DCFDA and flow cytometry. The results were expressed as a percentage of expression (fluorescencence intensity with respect to control) and as mean ± SD of three separate experiments. The largest effect was observed after 4 h of exposure. * *p* < 0.05 vs negative control

ROS were induced at all the tested concentrations at all tested times (10 min to 24 h). At 10 and 30 min after exposure, the first changes in redox status occurred (Fig. 4a and b). After 2 h, the redox statuses of the cells treated with lower concentrations of TiO_2_ NPs fell under the levels of the positive controls, and only concentrations of 10 μg/mL or higher induced shifts in redox status as large as those of the positive controls (*p* < 0.05 vs negative control) (Fig. 4c). After 4 h of exposure, all concentrations produced an effect above that of the positive controls in a concentration-dependent manner (*p* < 0.05 vs negative control) (Fig. 4d). After 8 h and 24 h, the shifts in redox statuses were equally large for all concentrations and were not different from that of the positive control (*p* < 0.05 vs negative control) (Fig. 4e-f).

**Fig. 5:**
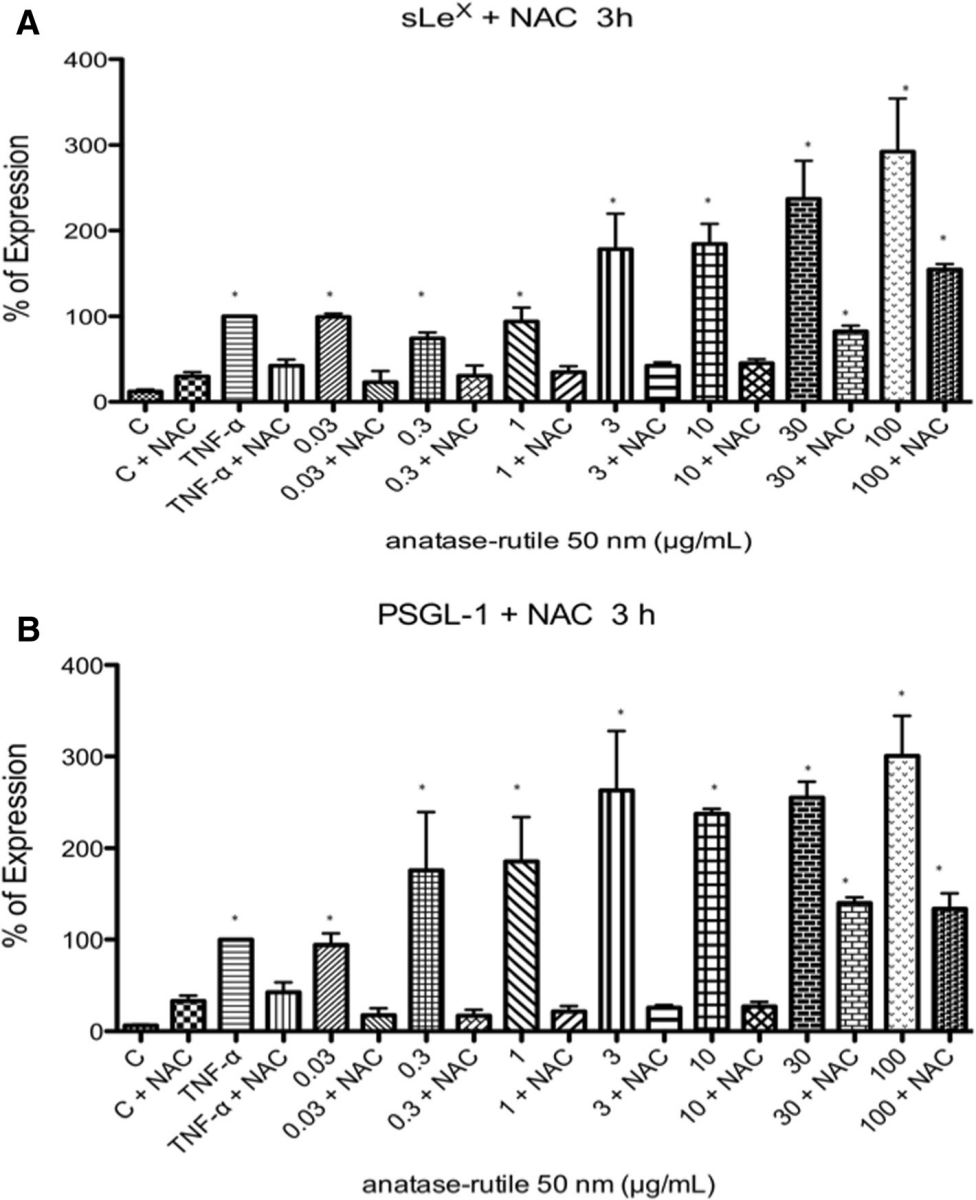
The role of ROS in the expression of early receptors for adhesion molecules was assessed using NAC. After 1 h of incubation of U937 cells with or without 10 μM of NAC, the cells were exposed to 0.03, 0.3, 0.1, 0.3, 1.0, 3.0, 10, 30 or 100 μg/mL of TiO_2_ NPs. The presence of NAC abolished the expression of sLe^x^ (**a**) and PSGL-1 (**b**) at concentration from 0.03 to 10 μg/mL. When the cells were exposed to 30 or 100 μg/mL, the reduction of sLe^x^ and PSGL-1 was partial in the presence of NAC

N-Acetil-Cystein (NAC decreased the expression of early adhesion molecules induced by TiO_2_ NPs. After exposure to TiO2 NPs during 3 h in the presence of NAC (10 μm) the expression of sLex and PSGL-1 were abolished from 0.03 to 10 μg/mL (Fig. 5). When exposed to 30 and 100 μg/mL of TiO2 NPs, the reduction of ROS by NAC was partial (Fig. 5).

**Fig. 6:**
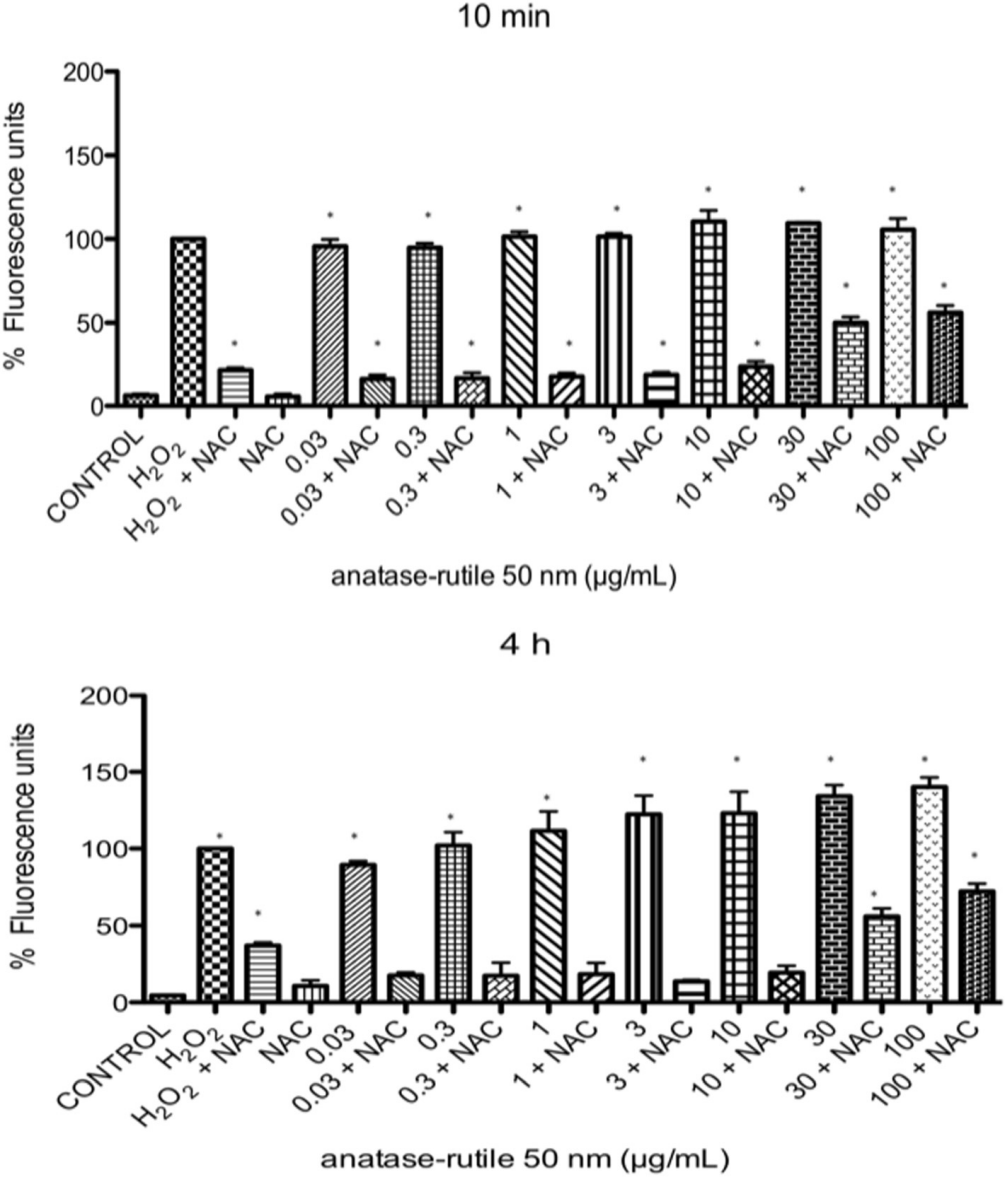
Evaluation of the ROS levels in U937 cells exposed to TiO_2_ NPs in the presence or absence of NAC. The presence of NAC in cultures of U937 cells exposed to TiO_2_ NPs abolished the levels of ROS, and only a partial reduction was observed if the cells were exposed to 30 or 100 μg/mL of TiO_2_ NPs

Oxidative stress was determined indirectly by measuring the production of H_2_O_2_ by means of H2DCFDA. After 10 min and 4 h of exposure to TiO_2_ NPs, a large amount of accumulated H_2_O_2_ was detected, however, it was inhibited when the cells were previously treated with NAC (Fig. 6). At concentrations of 30 and 100 μg/mL of TiO_2_ NPs, the inhibition of ROS by NAC was partial (Fig. 6).

## Discussion

Translocation and systemic effects induced by inhaled particles has been documented in recent years [10, 16], supporting epidemiological evidence that visits to emergency rooms after high levels of particulate matter air pollution increase due to cardiovascular complications after high levels of particulate matter pollution [16, 28, 29]. Regarding the vascular effects of inhaled particles, some authors have reported decreases in coagulation time [30], increases in the numbers of circulating clots [31] and evidence of endothelial dysfunction in distant vessels [32]. Our results show that monocytes exposed to very small concentrations of TiO_2_ NPs express molecules that are ligands for adhesion molecules and that these monocytes become adherent to naive endothelial cells. Some controversial results on the effects of NPs have been shown when ZnO was compared to TiO_2_, reporting that THP-1 cells exposed to ZnO increases the expression of MCP-1, migration and adhesion to HUVECs, but TiO2 was unable to induce similar effects [33].

Moreover, we found that HUVECs exposed to nanoparticles became adherent to U937 cells regardless of whether they were naive or NP-stimulated. These effects were observed at higher concentrations of NP. We may suppose that HUVECs treated with high concentrations of NP (50–100 μg/ml) lose their integrity due to shrinkage and cytoskeleton reorganization and become less adhesive [34]. TiO_2_ NPs is widely used [11], and despite the fact that there are not enough data on exposure levels, our results indicate that even if small quantities of these particles come into contact with monocytes, systemic effects may be induced.

Humans have always been exposed to particles by inhalation due to natural events or anthropogenic activities [35]. Despite the many efforts to reduce the amounts of particles in work places [36] and cities [37] within the last 50 years, the increased production of new nanosized materials and already known materials leaves open questions regarding the toxicity and controllability of these materials. The case of TiO_2_ is interesting due to its wide use in different industries [11]. The fine particles of TiO_2_ were considered as poorly soluble and with low toxicity. In some studies has been shown that reducing TiO_2_ to nanosize, is related to an increase of the surface area as well as changes in their electronic configuration and reactivity. Nowadays the fine particles are considered by the IARC as a 2B carcinogen, indicating that it is possibly carcinogenic for humans [12]. Other studies suggest that differences in the sizes, charges and crystalline structures of the particles may be critical in their toxicity [38]. In the present study we evaluated three different types and sizes of TiO_2_ NPs with different proportions of anatase and rutile (two main forms of TiO_2_). Because no difference was observed among the three types of TiO_2_ NPs evaluated, we focused only on the anatase-rutile with a size of < 50-nm, which was previously well characterized by us [17]. The results of the other two particles are presented as Additional file 1.

Vascular endothelium is the most important regulator of vascular homeostasis, both in physiological and pathophysiological conditions. Every alterations in endothelial functions are defined as ‘endothelial dysfunctions’, and these ‘dysfunctional’ processes have essential implications in the overall control of a vascular balance. Dysfunctional endothelium exhibit pro-thrombotic and proinflammatory activity which results in systemic response and vascular complications even in the peripheral regions of a vascular system [22–24]. It was clearly demonstrated that the prolonged and high dose pulmonary exposure of TiO_2_ NPs promotes the progression and vulnerability of atherosclerotic plaques in ApoE-knockout mice [19, 20]. The possible mechanisms involve the increased expression or secretion of pro-thrombotic (tissue factor, plasminogen activator inhibitor-1) and pro-inflammatory factors (ICAM-1, VCAM-1, monocyte chemoattractant protein-1, factors) and reduction in levels of benign regulators (serum high-density lipoprotein cholesterol - HDL, nitric oxide and tissue plasminogen activator – TPA) [20]. This deleterious effect of TiO_2_ is less significant or even moderate, if the dose and the time of exposure are reduced. Mikkelsen et al. demonstrated that repeated exposures to TiO_2_ NPs was associated with modest plaque progression in the same animal model [21]. However, ex vivo endothelium-dependent acetylcholine (ACh)-induced vasodilation of aorta segments from ApoE-knockout mice exposed by intratracheal instillation of TiO_2_ was significantly impaired in the presence of tempol (a free radical scavenger). This data suggest that locally generated free radicals contribute in endothelial dysfunction in its very primary phase [21].

Monocyte-endothelium interactions are mediated by different molecules promoting the capture, rolling, activation, adhesion, diapedesis and migration of monocytes [23]. Among these molecules, E, P and L selectins play an important role in capture and rolling events, whereas ICAM-1 and VCAM-1 play a main role in adhesion, and PECAM-1, in diapedesis [23]. Previously, we showed that endothelial cells exposed to urban PM_10_ and PM_2.5_ or TiO_2_ NPs express these molecules at concentrations of 5 to 40 μg/cm^2^ [17, 39, 40, 41]. When the exposed cells were co-cultured with naive human monocytes, the monocytes adhered to the endothelial cells. It has been shown that animals exposed to particles have large amounts of monocytes that adhere to the endothelium in vessels distant from the lungs [32]. Given that only very small amounts of inhaled particles may translocate to the blood and that particle concentrations may drop due to diffusion, it is unlikely that inhaled particles activate endothelial cells in distant vessels. Nevertheless, circulating monocytes may capture small amounts of particles, and if the expression of the ligands for adhesion molecules is upregulated, then adhesion to distant vessels seems plausible. In the present study, we demonstrated that human monocytes exposed to concentrations of TiO_2_ NPs as low as 0.03 μg/mL (30 ng/mL) increases the expression of sLe^x^ and PSGL-1 as early as 3 h (Fig. 1a-d), after exposure and LFA-1, VLA-4 and αVβ3 as early as 8 h after exposure (Additional file 1), reaching a peak 18 h after exposure (Fig. 2 a-f). We evaluated the adhesion of exposed monocytes to endothelial cells. If monocytes were co-cultured with naive endothelial cells, the adhesion was as strong as when the endothelial cells exposed to particles were co-cultured with naive endothelial cells (Fig. 3). Previous observations of monocytes adhering to distant blood vessels could be explained by the activation of monocytes coming into contact with very small amounts of translocating particles. Another proposed mechanism of nanoparticle transfer involves their redistribution within microvesicles released from activated macrophages. Intercellular trafficking of magnetic labeled nanoparticles has been observed between NP-stimulated macrophages and naïve monocytes [22]. Moreover it has been observed that the nanoparticles can be internalized within the cell; NPs first interact with the cell membrane and are subsequently internalized by vesicles, or by diffusion. Nevertheless in other repots it has been observed that nanoparticles are internalize into the cell, by other mechanisms such as phagocytosis and endocytosis.

When monocytes and endothelial cells were independently exposed to TiO_2_ NPs for 3 h and then co-cultured for another 3 h, the intensity of cellular adhesion was similar to that of previous experiments. The ability of TiO_2_ NPs stimulated endothelial cells to attach to monocytes may be evoked not only by the expression of adhesion receptors but also by changes in the physical properties of their cell surfaces, e.g., as seen after phosphatidylserine (PS) exposure. PS is normally restricted to the inner leaflet of the plasma membrane but in stressful conditions becomes exposed on the outer surface of viable endothelial cells and participates in macrovesicles shedding [42]. PS may activate monocytes and facilitate their adhesion to endothelial cells by an annexin-1-dependent mechanism [43]. In our study we did not investigate this interaction but cannot exclude the involvement of this specific mechanism.

Oxidative stress seems to play a central role in the effect of TiO_2_ NPs on monocytes. An initial large disruption of redox balance was observed after 10 min of exposure, which dropped after 30 min of exposure (Fig. 4). A more consistent concentration-response effect was observed after 2 and 4 h. After 8 and 24 h of exposure, the levels of redox balance were not different at the evaluated concentrations but were all as high as in the positive controls. Previous studies have shown the role of redox balance in the expression of the molecules we evaluated and therefore support our observations [44]. These results are interesting due to the role of the PSGL-1 involved in the development of several outcomes such thrombosis and cardiovascular diseases. In addition the PSGL-1 regulate the expression of other molecules involved in the inflammatory response. The interaction between the P-selectin and PSGL-1 activates the beta-2 integrin Mac-1 and the firm adhesion between the two cell types. The interaction of P-selectin with PSGL-1 also induces upregulation of leukocyte tissue factor, biosynthesis of several cytokines and other inflammatory reactions, thereby contributing to the thrombotic progression, therefore we suggest that the increase of PSGL-1 in the monocytes exposure could be implicated in development cardiovascular effects, however it is necessary to do more researcher of the role of PSGL-1 in the activation monocytes during the inflammatory process [45, 46].

## Conclusions

In conclusion, TiO_2_ NPs are capable of activating human monocytes at very low concentrations by increasing the expression of adhesion molecules ligands, which triggers monocyte-endothelial cell adhesion. These effects are dependent of the production of ROS.

## Methods

### Experimental design

Human cell line U937 was used as a monocyte cell model and HUVECs were used as a model for endothelial cells in the adhesion assays. Cells were exposed to 0.001, 0.003, 0.01, 0.03, 0.3, 1, 3, 10, 30 or 100 μg/mL of each type of TiO_2_ NPs. Unexposed cells were used as negative controls, and cells exposed to TNFα were used as positive controls. To evaluate the expression of sLe^x^, PSGL-1, LFA-1, VLA-4 and αVβ3, cells were exposed to TiO_2_ NPs for 3 to 24 h. The expression of different molecules was measured by flow cytometry using monoclonal antibodies labeled with FIT-C. All experiments were repeated at least three times. The role of ROS was evaluated by using NAC.

**Fig. 7:**
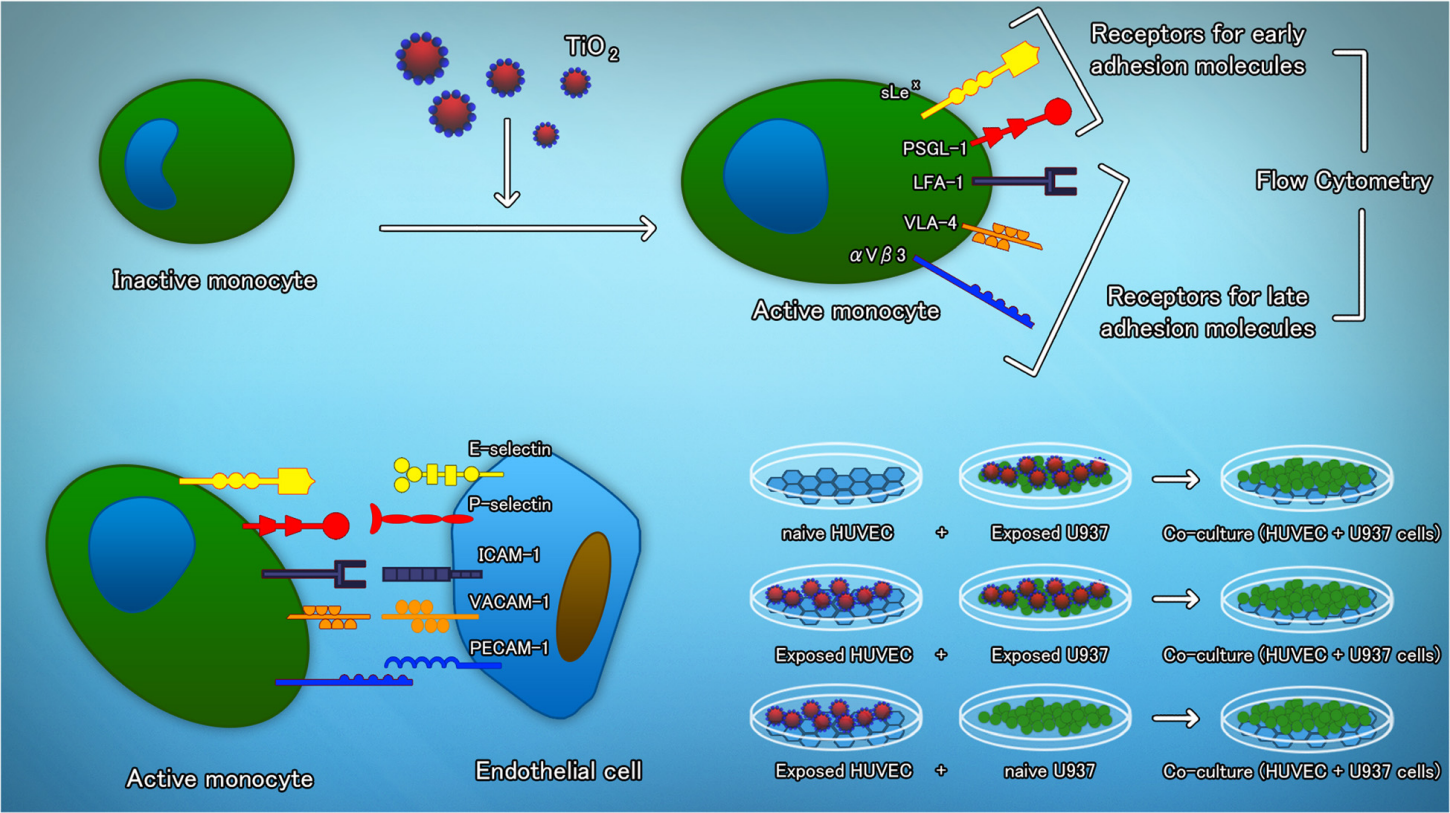
Schematic representation of the study design. Monocytes become activated after exposure to TiO_2_ NPs. The expression of early and late receptors for adhesion molecules was evaluated by means of flow cytometry. Different combinations of exposure to TiO_2_ NPs were used to evaluate if the monocytes become adherent to endothelial cells

The adhesion of monocytes to endothelial cells was evaluated under three different conditions as follows (Fig. 7): Monocytes labeled with [^3^H]-thymidine were exposed to 0.3, 1, 3.0, 10, 30 and 100 μg/mL of TiO_2_ NPs for 3 h and then added to naive endothelial cells. In the second approach, [^3^H]-thymidine labeled monocytes were exposed to the same concentrations of TiO_2_ NPs for 3 h and then added to previously HUVECs previously exposed to the same concentrations of TiO_2_ NPs. The third approach involved exposing HUVECs to TiO_2_ NPs concentrations and adding naive thymidine labeled monocytes after 3 h. After co-culturing both cell types during 3 h, the cultures were washed and then lysed. Unexposed cultures were used as negative controls, and cells exposed to TNFα (10 ng/mL) were used as positive controls.

#### Materials

RPMI 1640 and M199 media and trypsin were purchased from GIBCO/BRL (Grand Island, NY, USA), and fetal bovine serum (FBS) was purchased from HyClone (Logan, UT, USA). Sterile plastic material for tissue culture was purchased from NUNC and Corning. Flow cytometry reagents were purchased from Becton Dickinson, Immunocytometry Systems (San José, CA, USA). TNF$\alpha$ was purchased from R & D Systems (Minneapolis, MN, USA). A peroxidase-labeled monoclonal antibody against Von Willebrand factor was purchased from Santa Cruz Biotechnology (Santa Cruz, CA, USA). H2DCFDA was purchased from Molecular Probes.

##### Titanium dioxide nanoparticles

We used three different types of TiO_2_ NPs as follows: < 25 nm anatase (Sigma-Aldrich 637254) with a surface of 45–50 m^2^/g, 50 nm anatase-rutile [8] with a surface of 46 m^2^/g and 100 nm anatase-rutile (Sigma-Aldrich 634662). All particles were tested for the presence of endotoxin by a kinetic chromogenic Limulus Amebocyte Lysate (LAL). We used X-ray Fluorescence (XRF) analysis to determine if other elements (Al, Si, P, S, Cl, K, Ca, Ti, V, Cr, Mn, Fe, Ni, Cu, Zn, Se, Br, Sr, and Pb) were present in the particles. The 50 nm anatase-rutile used in this study was previously characterized by our work group [17].

The < 25 nm anatase and the < 100 nm anatase-rutile were evaluated by means of zetasizer (Zetasizer Nano ZSP, Malvern Instruments LTD, Worcestershire UK) and the mean particle size and ζ-potential was measured.

TiO_2_ NPs were handled under light-free conditions at all times. TiO_2_ NPs were sterilized by autoclave (1.5 atm, 20 min) the night before each experiment. Suspensions of TiO_2_ NPs in M199 medium (1 mg/mL), were prepared a few minutes before cell exposure. Aliquots were taken from these suspensions and further diluted with culture medium until the required final concentration was obtained. During the incubation periods, the exposed cells remained in the light-free conditions.

##### Culture of U937 Cells

Human leukemia promonocytic U937 cells were cultured in RPMI-1640 medium supplemented with 10 % FBS and L-glutamine (2 mM). We exposed 400,000 cells to 0.001, 0.003, 0.01, 0.03, 0.3, 1, 3, 10, 30 and 100 μg/mL of each type of TiO_2_ NPs or human recombinant TNFα (10 ng/mL).

##### Endothelial cell cultures

Primary human umbilical endothelial vein cell (HUVEC) cultures were obtained by proteolytic dissociation of umbilical cord veins from normal deliveries, treated with collagenase type II (0.2 mg/mL), and cultured on 0.02 % gelatin-coated culture dishes in M199 supplemented with 10 % FBS, glutamine (2 mM), heparin (1 mg/mL), and endothelial mitogen (20 μg/mL) as previously described [47]. Cells on their second passage were used for all experiments. The phenotype of the HUVEC cultures was confirmed by Von Willebrand antigen staining.

##### Evaluation of adhesion molecule receptor expression by flow cytometry

To evaluate the expression of sLe^x^ and PSGL-1, cells were exposed during 3, 6, 9, 12 or 24 h. To evaluate the expression of LFA-1, VLA-4 and αVβ3 cells were exposed for 3, 8, 12, 18 or 24 h. After these times, the cells were collected and centrifuged at 1200 rpm for 3 min. The cells were incubated with the different FITC-labeled monoclonal antibodies against human adhesion molecules receptor diluted 1:20. After 1 h of incubation, the cells were washed twice with PBS-albumin (8 % albumin and 0.02 % sodium azide), resuspended in 500 μL PBS, and immediately analyzed by flow cytometry (Fascalibur, Becton Dickinson). The results are expressed as percentages of expression compared with positive control cultures. To calculate the intensity of expression, we used the number of cells positive for FITC (FL1-H) multiplied by the mean of the fluorescence units (FU). The results for FU for the control cultures were considered 100 % and all the other data points were related to the positive controls to calculate the relative percentages of expression.

##### Adhesion of U937 cells to endothelial cells

Adhesion was evaluated using U937 cells labeled with [^3^H]-thymidine and exposed to 0.001, 0.003, 0.01, 0.03, 0.3, 1, 3, 10, 30 and 100 μg/mL of each type of TiO_2_ NPs for 3 h; 1 × 10^5^ HUVECs were seeded in 24-well tissue culture plates with 1 mL of supplemented M199 medium. Naive cultures were used to evaluate the adhesion of exposed monocytes. In other experiments, HUVEC were treated with TNF-α (10 ng/mL) or TiO_2_-NP, whereas 6 × 10^6^ U937 cells were incubated with 30 μCi of [^3^H]-thymidine for 48 h. HUVECs (naive or pretreated) were co-cultured with 5 × 10^5^ U937 cells/well (pretreated with TiO_2_-NP or naive) for 3 h. At the endpoint, each well was washed to eliminate any U937 cells not attached to the HUVECs, and the cells were fixed with 95 % methanol and lysed with NaOH (200 mM) for 12 h. Radioactivity was determined with a scintillation counter (Beckman Coulter model LS6500, Miami, FL, USA), and counts per minute (CPM) were considered directly proportional to the number of U937 cells adherent to the HUVECs.

##### Measurement of Reactive Oxygen Species (ROS) in U937 cells

The oxidation of 2, 7-dichlorodihydrofluorescein diacetate (H_2_DCFDA) into 2, 7-dichlorodihydrofluorescein (DCF) was used to assess ROS generation. U937 were cultured without or with three different types TiO_2_-NP (0.001, 0.003, 0.01, 0.03, 0.3, 1, 3, 10, 30 or 100 μg/mL) for 10, 30 min, 2, 4, 8 and 24 h. H_2_O_2_ (500 μM) was used as a positive control to induce ROS. After treatment, cells were incubated with H_2_DCFDA (10 μM) for 30 min at 37 °C and washed twice with PBS. After an extensive wash, fluorescence was evaluated by flow cytometry (Facscalibur, Becton Dickinson). The mean fluorescence intensity was calculated by multiplying the number of events (fluorescent cells) by the mean of the intensity presented by the Cell Quest software used for the analysis. Cultures exposed to H_2_O_2_ were considered as 100 % and all the other data points were related to the positive control to calculate the relative induction of ROS.

##### N-Acetil-Cisteína (NAC) and ROS production

U937 were cultured without or with NAC (10 μM) and TiO_2_ NPs (0.001, 0.003, 0.01, 0.03, 0.3, 1, 3, 10, 30 or 100 μg/mL) for 10 min and 4 h, and PM_10_ or in combination for 3 h. NAC was added 1 h before particles. H_2_O_2_ (500 μM) was used as positive control to induce oxidative stress. After treatment, cells were incubated with H_2_DCFDA (10 μM) for 30 min at 37 °C and washed twice with PBS. After extensive wash, fluorescence was evaluated by flow cytometry (Facscalibur, Becton Dickinson). The mean fluorescence intensity was calculated by multiplying the number of events (fluorescent cells) by the mean of the intensity presented by the Cell Quest software used for the analysis.

### Statistical analysis

Multiple comparisons were carried out by one-way ANOVA, followed by Fisher’s LSD multiple comparison test. Differences were considered statistically significant when *p* < 0.05.

## Abbreviations

CPM, counts per minute; FBS, fetal bovine serum; FU, fluorescence units; H_2_DCFDA, 2, 7-dichlorodihydrofluorescein diacetate; HUVECs, human umbilical vein endothelial cells; IARC, International Agency for Research on Cancer; NAC, N-acetylcysteine; ROS, reactive oxygen spicies; TiO_2_ NPs, titanium dioxide nanoparticles

## Additional file

Additional file 1: Results of the expression of early receptors for adhesion molecules at 6 and 9 h induced by TiO_2_ NPs with a size of 50 nm. Results of the expression of late receptors for adhesion molecules at 8 and 12 h induced by TiO_2_ NPs with a size of 50 nm. Results of viability of U937 cells exposed to TiO_2_ during 3, 6, 9, 12 or 18 h (0.001 to 100 μg/cm^2^). Results of the expression of early and late receptors for adhesion molecules induced by TiO_2_ NPs with a size of 25 nm (time-course, 3 to 24 h). Results of the expression of early and late receptors for adhesion molecules induced by TiO_2_ NPs with a size of 100 nm (time-course, 3 to 24 h). (DOCX 4681 kb)
