# Supplementary material for: Titanium dioxide nanoparticles induce the expression of early and late receptors for adhesion molecules on monocytes
Source: Part Fibre Toxicol. 2016 Jun 23;13:36. doi: 10.1186/s12989-016-0147-3 (PMC4917990; doi:10.1186/s12989-016-0147-3)
Supplement: Additional file 1: — Results of the expression of early receptors for adhesion molecules at 6 and 9 h induced by TiO2 NPs with a size of 50 nm. Results of the expression of late receptors for adhesion molecules at 8 and 12 h induced by TiO2 NPs with a size of 50 nm. Results of viability of U937 cells exposed to TiO2 during 3, 6, 9, 12 or 18 h (0.001 to 100 μg/cm2). Results of the expression of early and late receptors for adhesion molecules induced by TiO2 NPs with a size of 25 nm (time-course, 3 to 24 h). Results of the expression of early and late receptors for adhesion molecules induced by TiO2 NPs with a size of 100 nm (time-course, 3 to 24 h). (DOCX 4681 kb) [file 12989_2016_147_MOESM1_ESM.docx]

Titanium dioxide nanoparticles induce the expression of early and late receptor for adhesion molecules on monocytes.

Additional file 1

Induction of receptors for early adhesion molecules at concentrations from 0.001 to 100 μg/mL after 6 h of exposure to TiO_2_ NPs with a size of 50 nm

Induction of receptors for sLe^x^ early adhesion molecules at concentrations from 0,001 to 100 μg/mL after 6 h of exposure to TiO_2_ NPs with a size of 50 nm

100 µg/cm^2^

0.03µg/cm^2^

0.01µg/cm^2^

0.3µg/cm^2^

0.001µg/cm^2^

10 µg/cm^2^

3µg/cm^2^

0.003µg/cm^2^

30 µg/cm^2^

1µg/cm^2^

CONTROL

TNF-α

Induction of receptors for PSGL-1 early adhesion molecules at concentrations from 0,001 to 100 μg/mL after 6 h of exposure to TiO_2_ NPs with a size of 50 nm

10 µg/cm^2^

3µg/cm^2^

0.03µg/cm^2^

0.3µg/cm^2^

0.001µg/cm^2^

100µg/cm^2^

0.01µg/cm^2^

0.003µg/cm^2^

30 µg/cm^2^

1µg/cm^2^

CONTROL

TNF-α

Induction of receptors for early adhesion molecules at concentrations from 0.001 to 100 μg/mL after 9 h of exposure to TiO_2_ NPs with a size of 50 nm

Induction of receptors for sLe^x^ early adhesion molecules at concentrations from 0.001 to 100 μg/mL after 9 h of exposure to TiO_2_ NPs with a size of 50 nm

0.3µg/cm^2^

0.03µg/cm^2^

10µg/cm^2^

100 µg/cm^2^

30µg/cm^2^

0.01µg/cm^2^

0.001 µg/cm^2^

0.003µg/cm^2^

1 µg/cm^2^

TNF-α

CONTROL

3µg/cm^2^

Induction of receptors for PSGL-1 early adhesion molecules at concentrations from 0.001 to 100 μg/mL after 6 h of exposure to TiO_2_ NPs with a size of 50 nm

10µg/cm^2^

3µg/cm^2^

0.03µg/cm^2^

0.3µg/cm^2^

0.003µg/cm^2^

0.001 µg/cm^2^

100 µg/cm^2^

1µg/cm^2^

30 µg/cm^2^

0.01µg/cm^2^

CONTROL

TNF-α

Induction of receptors for late adhesion molecules at concentrations from 0.001 to 100 μg/mL after 8 h of exposure to TiO_2_ NPs with a size of 50 nm

* * * * * *

*

LFA-1

8 h

*

* * * * *

VLA-4

8 h

*

*

*

* * * * *

αVβ3

8 h

Induction of receptors for late adhesion molecules at concentrations from 0.001 to 100 μg/mL after 12 h of exposure to TiO_2_ NPs with a size of 50 nm

* * * * * * *

LFA-1

12 h

*

* * * *

* *

VLA-4

12 h

* *

* * * *

*

αVβ3

12 h

**Cristal Violet Staining.** Cell number was evaluated by crystal violet staining. U937 were cultured on 24-well plates without and with TiO_2_ NPs (0.001, 0.003, 0.01, 0.03, 0.3, 1, 3, 10, 30 and 100 to 10 μg/mL), for 3, 6, 9 12 and 18 h. Plates were washed three times by submersion in deionized water, air-dried, stained for 20 min with 400 *μ*L of a 0.1% crystal violet solution (in 200 mM phosphoric acid buffer at pH 6). After careful aspiration of the crystal violet solution, the plates were extensively washed with deionized water, air-dried prior to the solubilization of the bound dye with 250 *μ*L of a 10% acetic acid solution, and incubated during 30 min. Optical density of the plates was measured at 595 nm in a multiplate spectrophotometer.

Cell viability at concentrations from 0.001 to 100μg/mL after 3, 6, 9, 12 or 18 h of exposure to TiO_2_ NPs with a size of 50 nm

*

*

*

*

* * *

*

*

*

* *

* *

* *

* *

Induction of receptors for early adhesion molecules at concentrations from 0.001 to 100 μg/mL after 3, 6 or 9 h of exposure to TiO_2_ NPs with a size of 25 nm

*

Induction of receptors for late adhesion molecules at concentrations from 0.001 to 100 μg/mL after 8, 18 or 24 h of exposure to TiO_2_ NPs with a size of 25 nm

*

*

*

*

*

*

*

*

*

*

*

*

*

*

*

*

*

*

*

*

*

VLA-4

8 h

LFA-1

8 h

αVβ3

8 h

*

*

*

*

*

*

*

*

*

*

*

*

*

*

*

*

*

*

*

*

*

LFA-1

12 h

VLA-4

12 h

αVβ3

12 h

*

*

*

*

*

*

*

*

*

*

*

*

*

*

*

*

*

*

*

*

*

LFA-1

24 h

VLA-4

24 h

αVβ3

24 h

Induction of receptors for early adhesion molecules at concentrations from 0.001 to 100 μg/mL after 3, 6 or 9 h of exposure to TiO_2_ NPs with a size of 100 nm

Induction of receptors for late adhesion molecules at concentrations from 0.001 to 100 μg/mL after 8, 18 or 24 h of exposure to TiO_2_ NPs with a size of 100 nm

*

*

*

*

*

*

*

*

*

*

*

*

*

*

*

*

*

*

*

*

*

LFA-1

8 h

VLA-4

8 h

αVβ3

8 h

*

*

*

*

*

*

*

*

*

*

*

*

*

*

*

*

*

*

*

*

LFA-1

18 h

VLA-4

18 h

αVβ3

18h

*

*

*

*

*

*

*

*

*

*

*

*

*

*

*

*

*

*

*

*

*

LFA-1

24 h

VLA-4

24 h

αVβ3

24 h
